# Carpal Dimensions by Plain Wrist Radiography in Patients with Severe Carpal Tunnel Syndrome

**DOI:** 10.1155/2022/1517057

**Published:** 2022-03-30

**Authors:** Seyyed Houssein Saeed-Banadaky, Hossein Rahimian, Mohammad Reza Sobhan

**Affiliations:** Shahid Sadoughi University of Medical Science and Health Services, Orthopedic Devision, Yazd, Iran

## Abstract

In this study, we evaluated the diagnostic value of carpal dimensions in wrist plain radiography for the screening of carpal tunnel syndrome (CTS). This is a case-control diagnostic probe in which patients with severe CTS documented by electrodiagnostic study and healthy subjects as controls were enrolled. In the posteroanterior view of the wrist plain radiography in both groups, we defined and measured the carpal ratio, and the results were analyzed deploying statistical software. In this study, 119 participants, including 50 patients and 69 healthy subjects, were recruited. According to the ROC chart, the cutoff points, positive and negative predictive values, and the diagnostic accuracy for the cutoff points were calculated.

## 1. Introduction

Carpal tunnel syndrome (CTS) is the most common entrapment neuropathy in extremities [1], which is caused by the compression of the median nerve in the wrist. CTS diagnosis is based on the clinical signs and symptoms and is confirmed by electrodiagnostic studies [2]. Because electrodiagnostic studies are invasive procedures, other diagnostic modalities such as sonography are used for the purpose. There are controversial results concerning the usefulness of ultrasound findings for evaluation of CTS [3–5]. Moreover, MRI and anthropometric measurements are used for diagnosis and prediction of the treatment [6, 7]; however, they cannot be replaced for EDS as a gold standard diagnostic test. Recently, digital X-ray machines have proven popular and medical imaging softwares can measure decimal distances in digital images. We attempted to conduct a case-control study to determine whether the measurement of carpal bone dimensions by a digital X-ray can predict severe CTS; in case the results proved positive, the technique could be used for the screening patients suspected of CTS along with the physical exam findings.

## 2. Materials and Methods

### 2.1. Methods

In this case-control study, we included all severe cases of CTS predicated on physical examinations and electrodiagnostic findings. The sample size was calculated according to the CTS estimated prevalence in our society population [8]. The subjects were selected out of the outpatients affected with upper extremity symptoms visiting the subspecialty polyclinic of the area affiliated to a general hospital from January 2019 to January 2020.

Cases with a history of metabolic or rheumatologic diseases, trauma, or any operation in the wrist as well as the mild and moderate cases of CTS were excluded.

The participants in the control group were selected from among adult volunteers without CTS risk factors and those with no history of CTS.

**Figure 1:**
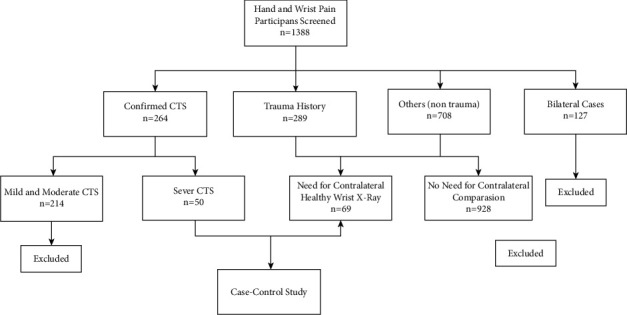
Flowchart of patient selection.

A single assessor took a careful history and examined the cases. The hypothesis outlined here was based on the relationship between carpal tunnel length/width ratio and the likelihood of severe CTS. Accordingly, carpal length in the axis of the 3rd ray (the distance between the proximal of lunate bone to the third metacarpal base), carpal width (the shortest distance between the medial part of trapezium tubercle to the most lateral part of hamate hook), and carpal ratio (calculated by dividing carpal length to carpal width) were considered as the radiographic criteria, respectively (Figure 1). A researcher-designed checklist was deployed to collect demographic information including age and sex. The standard PA wrist digital radiography was performed for patients and controls. Radiography was conducted in digital imaging center of the hospital. The patients were finally assigned to two groups (cases and controls). A single operator measured the criteria using Macropacs (Tahavolat Novin Yademan, Tehran, Iran) medical imaging software.

### 2.2. Statistical Analysis

The data were analyzed using SPSS software (version 22). Sensitivity, specificity, positive predictive value (PPV), negative predictive value (NPV), and accuracy of diagnosis were calculated. Then, the ROC curve was drawn to calculate the area under the curve for investigating the best CTS diagnostic point by length/width ratio of the wrist bones. Cutoff point was determined for CTS diagnosis by length/width ratio of the wrist bones using the greatest value obtained by multiplying the sensitivity and specificity. Concerning ethical dimension, radiography proved safe for the patients in this study. The research was conducted once the permission was issued by the research council of the university, and informed consent was obtained from the participants. The patients were then ensured in terms of confidentiality.

### 2.3. Ethical Approval

Ethical approval for this study was obtained from Institutional Research Ethics Committee School of Medicine, Shahid Sadoughi University of Medical Sciences (IR.SSU.MEDICINE.REC.1395.205).

### 2.4. Informed Consent

Written informed consent was obtained from all subjects before the study.

## 3. Results

**Figure 2:**
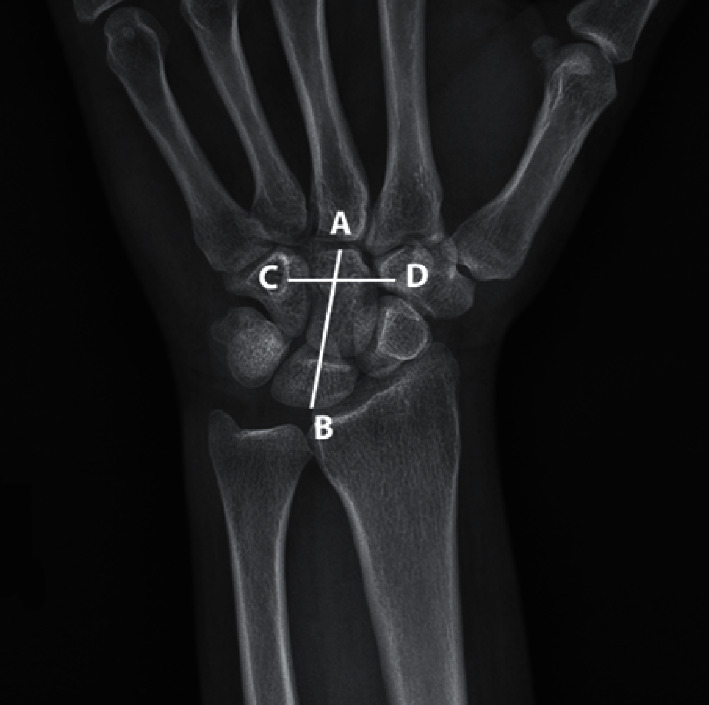
Wrist radiographic criteria. AB, carpal length. CD, carpal width.

This study was conducted from January 2019 to January 2020. During the study period, 1388 patients with symptoms of hand and wrist pain were referred. Based on paraclinical examinations, 264 patients were diagnosed with CTS. Among these patients, 214 had mild-moderate symptoms. These patients were excluded from the study. The remaining 1124 patients had the history of recent trauma or hand pain without a history of trauma. Among these patients, 127 were excluded due to their bilateral nature. Of the remaining patients, 69 required X-ray imaging to compare radiology of the wrist with the normal wrist on the opposite side, which was performed and included in the study (Figure 2). So, 50 were diagnosed with severe CTS using clinical exam and electrodiagnostic studies and 69 were found to be healthy. As given in Table 1, totally, 28 patients (23%) were male and 91 (77%) were female; in the experiment group, however, 10 were male and 40 female. The mean age in case and control groups turned out to be 45.3 (SD 9.7) and 42.6 (SD 12.0), respectively. Data were then analyzed by the *t*-test, the results of which indicated no statistical difference between the mean ages of the groups.

The mean of carpal length, carpal width, and carpal ratio for cases and controls are given in Table 2. Carpal length and carpal width in univariable and multivariable status are given in Table 3. Based on this table, carpal width had a significant difference in univariable and multivariable status.

## 4. Discussion

Electrodiagnostic studies remain the gold standard method for diagnosis of CTS [2]. However, due to the invasiveness of the method, the value of other diagnostic methods has been investigated. A host of authors have examined the value of MRI, ultrasound, radiography, and anthropometric measurements for diagnosis or screening of cases. The basis for all these studies is the measurement of the wrist carpal bone and median nerve dimensions as well as their relationship. The accuracy of MRI for diagnosis and the grading of CTS has been approved in some studies [3, 9], but this is a relatively expensive procedure for routine examinations.

Bleecker measured the cross-sectional area of the carpal tunnel using CT scan and finally identified cross-the section area in CTS patients being significantly lower than that of the control group [9].

**Figure 3:**
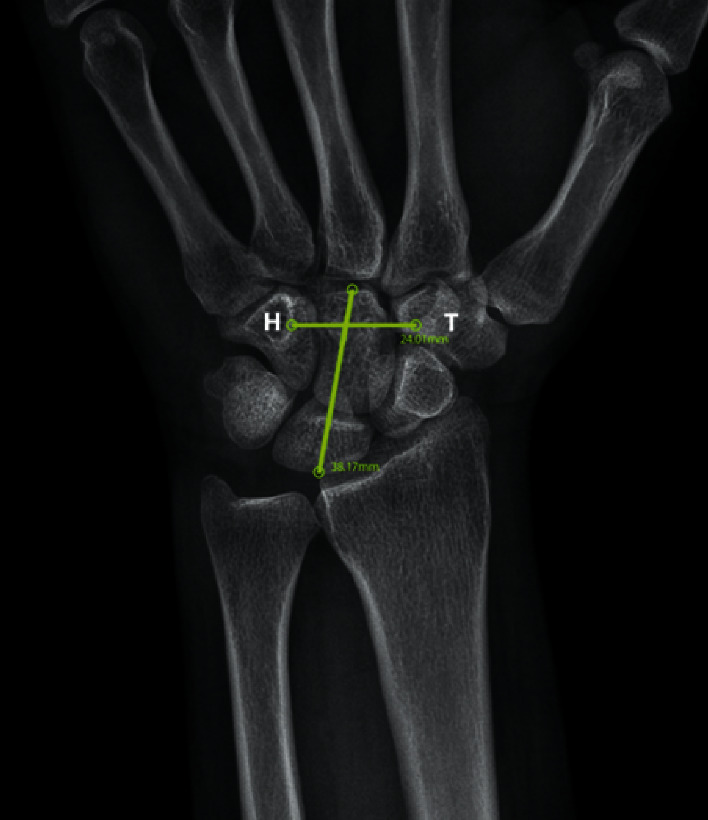
Decimal measurement of carpal tunnel dimensions by digital radiography. H, hamate; T, trapezium.

Recently, ultrasound is increasingly used for the diagnosis of CTS. By measurement of the cross-section area of the median nerve in a different area of the patients' wrist and compression with a control group, Bagga uncovered that ultrasound can be beneficial for diagnosis and grading of CTS [7]. Drakopoulos examined 96 patients after which they proved that the cross-section area of the median nerve at the pisiform level measured by the ultrasound can be as valuable as electrodiagnostic studies [1]. Borire reported that the ultrasound can detect the syndrome in borderline or even false negative cases diagnosed by electrodiagnostic studies [10]. On the other hand, De Kleermaeker detected low sensitivity for the ultrasound, so that it cannot replace electrodiagnostic studies for diagnosis of CTS [11]. Some researchers have employed plain X-ray as a noninvasive way for the evaluation of CTS patients. X-ray is known as a popular and low-cost tool; however, nowadays, digital X-ray machines and their software have made it possible to make more accurate measurements of radiographic dimensions on a decimal scale (Figure 3).

Much as the diagnosis of CTS by plain X-ray is unimaginable, finding any relationship between the syndrome and X-ray parameters can help screening of CTS. Bindra et al. investigated 447 wrist X-ray in cases with CTS and only in two cases, found radiographic changes with therapeutic significance [12]. They concluded that plain X-ray does not seem to be necessary for routine evaluation of the patients. Ikeda et al. [13] evaluated 94 wrists in 62 CTS patients and 94 asymptomatic normal wrist by X-ray; they measured the volar tilt, radial inclination, ulnar variance, transverse, and anteroposterior distance at the distal radius. These researchers finally identified a positive correlation between ulnar variance and CTS and concluded that a positive ulnar variance should be considered a risk factor for developing CTS. They did not, however, measure the dimensions of the carpal tunnel directly and used the pure lengths rather than rates. We believe the measurement of rates being more valuable than pure length as for the variety in skeletal size of the human being. We used the carpal ratio (CR) that is carpal height divided by carpal width (Figures 1 and 3) measured in digital radiography provided by Macropacs (Tahavolat Novin Yademan, Tehran, Iran) software. We checked the carpal width at the level of the hamate hook because this is the narrowest site of the carpal tunnel [5, 14]. Furthermore, we included severe cases of the disease in the study to expand it to all cases in the second phase of the study if a logical relationship could be found. After calculating CR in cases and control groups, the data were analyzed by software. Based on the ROC chart for carpal ratio, two cutoff points at CR = 1.32 and CR = 1.33 were obtained with a higher sensitivity and better positive and negative predictive value for CR = 1.32 (Figure 2 and Table 2). Despite developing this cutoff point by data analysis, the ROC curve pattern with two peaks is unusual and indicates heterogeneous data. Moreover, AUROC that was 0.660 indicates that our CR index cannot be a good and worthy test for detecting CTS [15]. Although the measurement of the cross-section area in the carpal tunnel by ultrasound and computerized tomography in some studies has a relationship with CTS, we are unable to show any correlation between the two-dimensional index of CR and CTS. We hope that some developments in radiological softwares, 3D, and volumetric measurement will be possible by plain X-ray so that we gain the possibility to use them for screening and detecting the risk factors for CTS. We failed to assess the intraclass correlation coefficient for measurement to build a weak point for our study; however, because of our negative results, it does not seem to matter.

## 5. Conclusion

The electrodiagnostic method remains to be the gold standard for CTS diagnosis. While it is true that our index in plain wrist radiography, named carpal ratio, bears a good sensitive rate of 82% in CR = 1.32, it cannot be used for screening of cases due to other statistical parameters mentioned earlier. On the basis of the studies that found a decrease in the cross-section area of the tunnel, although the etiology of CTS can be an elevated volume of soft tissue contents of the tunnel, we recommend conducting some other studies based on the measurement of carpal tunnel distances in three dimensions by digital X-ray deployed as a cheap and popular device.

## Figures and Tables

**Table 1:** Frequency distribution of the groups based on sex and age.

Variable		Case	Control
Sex (*N*)	Male	10 (20%)	18 (26%)
	Female	40 (80%)	51 (74%)
Age (mean, year)		45.32	42.60

**Table 2:** Comparison of case and control groups based on carpal length, carpal width, and carpal ratio.

Variable	Case	Control	*P* value
Carpal length (mean ± SD)	29.1300 ± 2.96842	37.3145 ± 3.70220	<0.001
Carpal width (mean ± SD)	21.0000 ± 2.14333	27.4304 ± 2.26224	<0.001
Carpal ratio (mean ± SD)	1.3905 ± 0.09451	1.3620 ± 010088	0.121

**Table 3:** Carpal length and carpal width (univariable and multivariable) and the two dichotomized carpal ratios (cutoff points 1.32 and 1.33).

Cutoff points	Variables	Univariable		Multivariable^*∗*^	
		OR, 95% CI	*P* value	OR, 95% CI	*P* value
1.32	Carpal length	1.02 (0.94, 1.10)	0.587	1.04 (0.96,1.13)	0.322
	Carpal width	0.82 (0.73, 0.93)	0.002	0.84 (0.75,0.95)	0.006

1.33	Carpal length	1.04 (0.97,1.13)	0.239	1.06 (0.98,1.15)	0.113
	Carpal width	0.87 (0.78,0.97)	0.010	0.88 (0.79,0.98)	0.026

^*∗*^Adjuster on age and gender.

## Data Availability

The data used to support this study are included within the article.

## Abbreviations

CTS: Carpal tunnel syndrome
ES: Electrodiagnostic studies
PPV: Positive predictive value
NPV: Negative predictive value
ROC: Receiver operating characteristic.
